# Does the rattle of *Crotalus durissus terrificus* reveal its dietary history?

**DOI:** 10.1186/1678-9199-20-53

**Published:** 2014-12-09

**Authors:** Melissa Gaste Martinez, Carlos Ducatti, Evandro Tadeu Silva, Savio Stefanini Sant’Anna, Maria Márcia Pereira Sartori, Benedito Barraviera

**Affiliations:** Center for the Study of Venoms and Venomous Animals, São Paulo State University (UNESP – Univ Estadual Paulista), Botucatu, São Paulo State Brazil; Stable Isotopes Center (CIE), Botucatu Biosciences Institute, São Paulo State University (UNESP – Univ Estadual Paulista), Botucatu, São Paulo State Brazil; Laboratory of Herpetology, Butantan Institute, São Paulo, São Paulo State Brazil; Botucatu Medical School, São Paulo State University (UNESP – Univ Estadual Paulista), Botucatu, São Paulo State Brazil; CEVAP/UNESP, Rua José Barbosa de Barros, 1780, Fazenda Experimental Lageado, Botucatu, SP CEP 18610-307 Brazil

**Keywords:** Food, Carbon-13, *Crotalus durissus terrificus*, Stable isotopes

## Abstract

**Background:**

Environmental devastation threatens the survival of many species, including venomous snakes such as the South American rattlesnake *Crotalus durissus terrificus*. This observation is based on the decrease of snakes collected and donated to Brazilian research institutes. Nevertheless, some individuals have managed to survive and procreate. The question is how these snakes are adapting in these new environmental conditions.

**Methods:**

To answer it, the carbon-13 level of rattlesnakes and their feed (either laboratory or wild mice) was evaluated by isotope-ratio mass spectrometry. Thus, rattle segments from 16 adults and 15 offspring of captive snakes, and of three wild newborn *C. d. terrificus* were evaluated as well as 17 *Mus musculus* mice captured in traps, four live feeder mice and the ration offered to mice at animal houses.

**Results:**

The isotopic exchange time of the captive adult snakes (n = 16) varied between 33 and 37 months and of captive-born animals (n = 15), until reaching a plateau of equilibrium, varied from 18 to 24 months. Regarding the captured *Mus musculus* (n = 17), 88.23% (n = 15) were from a C_4_ environment. Of the six rattle rings from offspring of captured *C. d. terrificus*, five were from a C_4_ environment, whereas of the 170 rattle rings studied, 60% originated from a C_3_ environment and 40% from a C_4._ The same carbon-13 values were found in captive snakes.

**Conclusions:**

Based on the present results, it can be inferred that most *C. d. terrificus* snakes (60%) fed animals from a C_3_ environment; birds consist of an alimentary alternative for snakes, as well as rodents, small reptiles and amphibians; different venom compositions among snakes from the same region may be related to the food type; the primary rattle of offspring reflects the maternal diet during gestation; and, finally, the different rattle rings indicate the alimentary history of these animals.

**Electronic supplementary material:**

The online version of this article (doi:10.1186/1678-9199-20-53) contains supplementary material, which is available to authorized users.

## Background

The South American rattlesnake *Crotalus durissus terrificus*, characterized by a caudal rattle, has proven to be able to procreate and survive in hostile environments. Once born, the offspring carry at the caudal tip a single ring denominated the primary rattle. At each molting, which generally occurs every 6 to 12 months depending on the diet, a new ring is formed. Throughout the life of these animals, they accumulate numerous rings, thus augmenting their rattle permanently. Environmental devastation requires these animals to adapt to new living conditions. Although the number of human accidents has increased in certain regions of Brazil, the number of specimens captured and donated to research institutes has diminished [1–4]. The question is how are these snakes adapting to and surviving in new environmental conditions?

To answer this query, the authors analyzed the isotope carbon-13 (δ ^13^C) of rattle segments from young and adult *C. d. terrificus* rattlesnakes as well as of food offered to them by means of mass spectrometry [5, 6]. This research was based on the knowledge that plants around the globe present different biochemical pathways, by absorbing molecules in their stable isotopes, especially carbon (^12^C, ^13^C), hydrogen (^1^H, ^2^H), oxygen (^16^O, ^17^O, ^18^O), nitrogen (^14^ N, ^15^ N) and sulfur (^32^S, ^33^S, ^34^S, ^36^S). During photosynthesis, when carbon fixation results in three-carbon atoms, the process is called C_3_ photosynthetic plant cycle. When the initially formed sugars have four carbon atoms, the process is called C_4_ photosynthetic plant cycle. Group C_3_ comprise approximately 86% of plant species, including hardwoods, pine and eucalyptus forests, rice, soy, other leguminous and fruit-bearing trees. C_4_ plants comprise grasses, corn and sugarcane (see Additional file 1) [7].

According to Vogel [7], the isotopic ratios and δ^13^C‰ values in C_3_ and C_4_ plants are expressed in the Pee Dee Belemnite (PDB) standard. The standard was named after a Cretaceous marine fossil, *Belemnitella americana*, found in the Pee Dee Formation in South Carolina, USA. This material had an anomalously high ^13^C:^12^C ratio (0.0112372), and was established as δ^13^C value of zero. The δ^13^C‰ of C_3_ plants varies from −22‰ to −33‰ with an average value of −26.7‰ whereas that of C_4_ plants ranges from −9‰ to −16‰, determining an average value of −12.6‰ and sample mean of −12.5‰. According to Martinez [8], environments that present intermediate values between −16‰ and −22‰ can be classified as partially C_3_ or C_4_. Thus, values between −16‰ and −17.2‰ indicate partially C_4_ plants and between −22‰ and −23.23‰ would suggest partially C_3_ plants. Thus, the isotopic variations of δ^13^C‰ found in organic matter may be used in the quality control, authentication and traceability of products originating from C_3_ and C_4_ plant environments [7].

The objective of this work was to evaluate the levels of (δ^13^C) in the rattle rings of *C. d. terrificus* snakes captured and born in captivity, in captured and laboratory mice, and in the ration offered to mice for diagnosing whether the alimentary history of the snakes is related to the C_3_ or C_4_ environment or both.

## Methods

### Captivity study

Captive animals were donated by the Center for the Study of Venoms and Venomous Animals (CEVAP) of São Paulo State University (UNESP) based in Botucatu, SP, Brazil (latitude 22° 53′ 09“ South, longitude 48° 26′ 42” West, and average altitude 804 meters) and by the Butantan Institute (IB) in São Paulo, Brazil (latitude 23° 32′ 51“ South, longitude 46° 38′ 10” West and altitude 760 meters).

We evaluated segments of rattles of 16 adult *C. d. terrificus* snakes kept in captivity (11 at CEVAP and five at IB) and 15 newborn snakes (four born in CEVAP and 11 in IB). Four mice (two from the CEVAP animal house and two from IB) and samples of feed offered to rodents reared in IB and CEVAP were studied.

### Field study

A field study was conducted in the city of Pardinho, about 20 km from Botucatu (latitude 23° 4′ 52“ South, longitude 48° 22′ 25” West and average altitude 898 meters) where most of the snakes donated to CEVAP were captured. At this site, pitfall traps were placed to capture mice, small rodents and newborn snakes [9, 10]. Three newborn *C. d. terrificus* and 17 *Mus musculus* mice captured in these traps in Pardinho were studied.

### Collection of material

The rattle of each *C. d. terrificus* snake was manually removed, then counted and placed into a tube of transparent plastic. The rattles were separated and identified one-by-one according to the origin of the animal. In total, 34 rattles were collected and 170 rings analyzed.

Captured mice and those provided by IB and CEVAP were killed according to the guidelines of the Ethics Committee on Animal Experimentation. Authorization for the field study was obtained from the Brazilian Institute of Environment and Renewable Natural Resources (IBAMA) – authorization for scientific purpose (number 13999–1).

### Isotopic analysis

For measurement of the isotopic ratio of carbon-13 (δ^13^C) in snake rattles, mice and their ration, three samples weighing from 50 to 70 μg were inserted into capsules maintained in a refrigerator at +4°C, in the laboratory of the Stable Isotopes Center of UNESP, Botucatu, SP. The employed methodology was proposed by Tieszen *et al*. [11], with adaptations by Ducatti *et al.* [6]. The isotopic ratios of ^13^C/^12^C were measured in a Delta V Advantage Isotope Ratio Mass Spectrometer (Thermo Fisher Scientific, USA). Isotopic ratio values were expressed as delta per thousand (δ) relative to the PDB international standards for ^13^C.

### Statistical analysis

The analysis and δ^13^C curve values obtained for each animal were obtained statistically by the Minitab method of regression for non-linear equations [12].

### Ethics committee approval

This study was approved on April 29, 2008 by the Brazilian Institute of Environment and Renewable Natural Resources (IBAMA) – SISBIO (authorization for activities with scientific purposes) under protocol number 13999–1.

## Results

By analyzing the samples and diet of rodents provided by CEVAP and IB, the following results were obtained: the average isotopic values of δ^13^C of rodents from CEVAP and IB animal house were respectively −21.46‰ ± 0.15‰ and −22.03‰ ± 0.07‰; for the ration given to rodents, the respective mean values for CEVAP and IB were −21.56‰ ± 0.08‰ and −22.56‰ ± 0.02‰.

**Figure 1:**
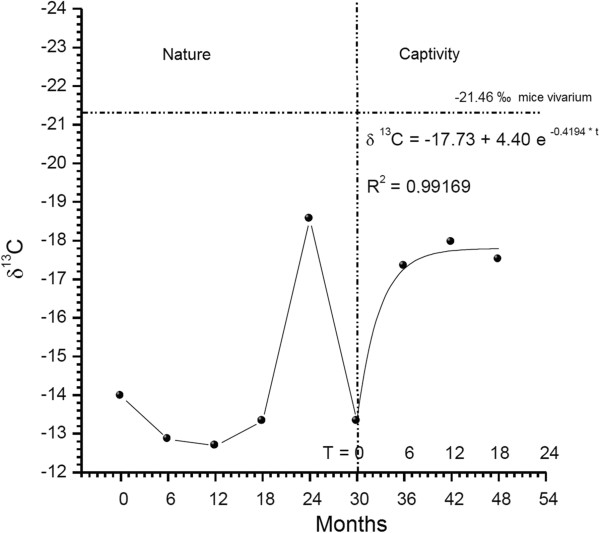
**Values of δ** ^**13**^ **C of the nine segments of the rattlesnake 1 from CEVAP.**

**Figure 2:**
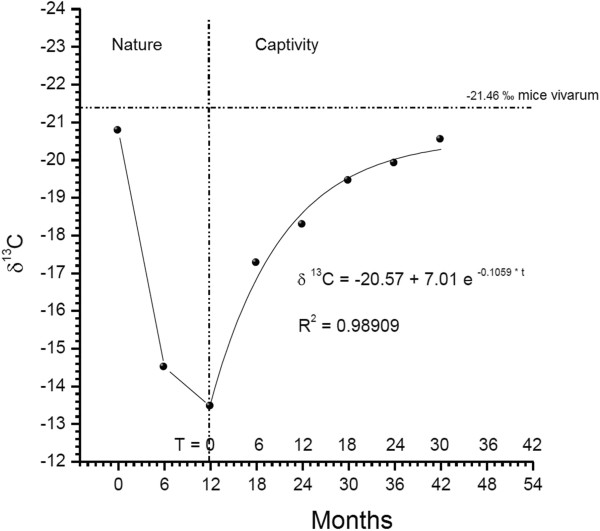
**Values of δ** ^**13**^ **C of the eight segments of rattlesnake 4 from CEVAP.**

**Figure 3:**
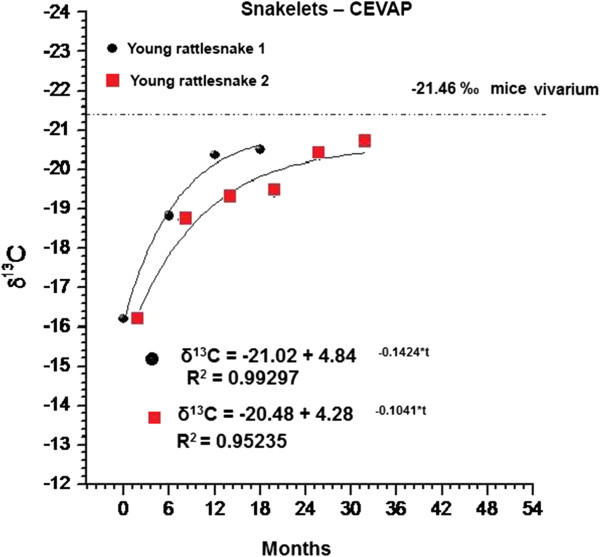
**Values of δ** ^**13**^ **C of rattle segments from two young snakes born in CEVAP captivity.**

We chose the results of the δ^13^C profile from two out of the 16 adult snakes (called “rattlesnake 1” and “rattlesnake 4”) captured in nature and maintained at CEVAP with, respectively nine and eight rings in the rattles (Figures 1 and 2). Of the four young animals born at CEVAP, we chose two (called “young rattlesnake 1” and “young rattlesnake 2”) with respectively four and six rings in the rattle (Figure 3).

Figure 1 shows the analysis of the δ^13^C results obtained from the nine segments of rattlesnake 1, from Botucatu, captured in a maize crop and kept for at least 24 months in CEVAP. The analysis revealed isotopic δ^13^C values of −13.98‰ for the first segment, −18.56‰ for the fifth and −13.28‰ for the sixth segment. The results observed in the seventh, eighth and ninth segments were respectively −17.34‰, −17.96‰ and −17.51‰. Figure 1 shows these results.

Figure 2 shows the analysis of the δ^13^C results obtained from the eight segments of rattlesnake 4, which was kept in captivity at CEVAP. Its respective δ^13^C isotopic values were −20.77‰ for the first segment, −14.5‰ for the second and −13.46 ‰ for the third segment. This snake was held in captivity for at least 42 months, where it received a diet of mice bred in CEVAP. The results observed in the fourth, fifth, sixth, seventh and eighth segments were respectively −17.26‰, –18.28‰, −19.44‰, −19.9‰ and −20.53‰, as shown in Figure 2.

Figure 3 shows the δ^13^C results obtained from the four segments of the young rattlesnake 1 and six segments from the rattle of the young rattlesnake 2, both born in CEVAP. For the young rattlesnake 1: the value of first segment was equal to −16.2‰. The results of the second, third and fourth segments were respectively −18.83‰, −20.37‰, and −20.52‰. The respective values for the young rattlesnake 2 were: −16.07‰, −18.61‰, −19.15‰, −19.26‰, −20.23‰ and −20.57 ‰ for the first, second, third, fourth, fifth and sixth segments, as displayed in Figure 3.

**Figure 4:**
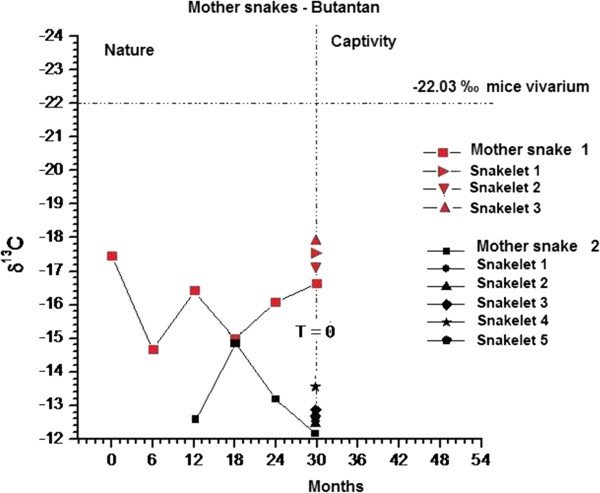
**Values of δ** ^**13**^ **C of the rattle segments of the two mothers and their offspring, all maintained in IB.**

For this study, the Butantan Institute donated two pregnant adult snakes (called “mother rattlesnake 1” and “mother rattlesnake 2”) kept in captivity who delivered eight offspring. The first and second had three and five offspring respectively, as shown in Figure 4.

The δ^13^C results obtained from the six segments of the mother rattlesnake 1 were as follows: the first segment showed a δ^13^C isotopic value of −17.45‰, whereas the second, third, fourth, fifth and sixth produced respective percentages of −14.67‰, −16.38 ‰, −14.98 ‰, −16.09‰ and −16.62‰. When this snake was captured and taken to IB, it had six segments in its rattle. One month later it gave birth to three snakelets whose δ^13^C values measured in the first segments were respectively –17.55‰., −17.09‰ and −17.90‰. The mother rattlesnake 2, held captive for a month at IB, had the respective δ^13^C values for its segments: −12.65‰, −14.97‰, −13.26‰ and −12.21‰. The five snakes born in captivity showed δ^13^C values for the primary/first segment of −12.59‰, −12.51‰, –12.94‰, −13.65‰ and −12.72‰. Figure 4 displays the results.

**Figure 5:**
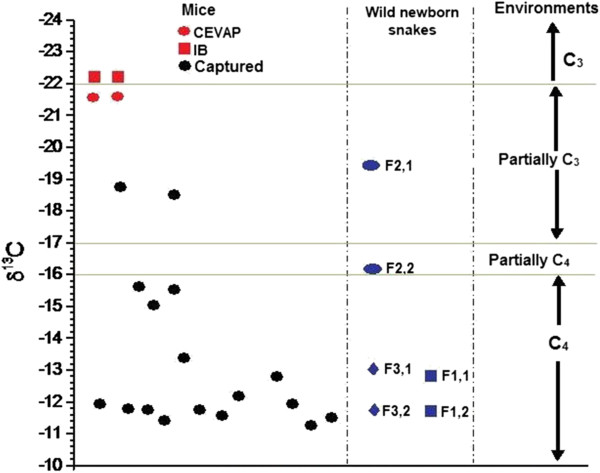
**Values of δ** ^**13**^ **C in mice bred in CEVAP and IB animal houses, in 17 mice captured in the field study and in segments of rattle of the three newborn captured snakes according to the environment (C**_**3**_ **, C**_**4**_ **and partially C**_**3**_ **and C**_**4**_ **).** F1,1 and F1,2: first wild newborn snake, primary and second segments; F2,1 and F2,2: second wild newborn snake, primary and second segments; F3,1 and F3,2: third wild newborn snake, primary and second segments.

In Figure 5, we analyzed the values of δ^13^C in two mice bred at CEVAP, two at IB, 17 captured in the field study and in segments of three wild newborn *C. d. terrificus*, according to the environmental distribution.

The mice bred in CEVAP and IB animal houses showed respective δ^13^C averages of −21.46 ± 0.15‰ and −22.03 ± 0.07‰. The two mice caught in the partially C_3_ environment showed average δ^13^C values of −18.73 ± 0.07‰, whereas the fifteen mice captured in the C_4_ environment presented a δ^13^C average of −11.92 ± 0.05‰. For the primary and second segments, the second wild newborn (F2) presented respective δ^13^C values of −19.57‰ and −16.57‰ versus −12.57‰ and –11.50‰ for the first wild newborn (F1) and −13.89‰ and −11.59‰ for the third wild newborn (F3).

**Figure 6:**
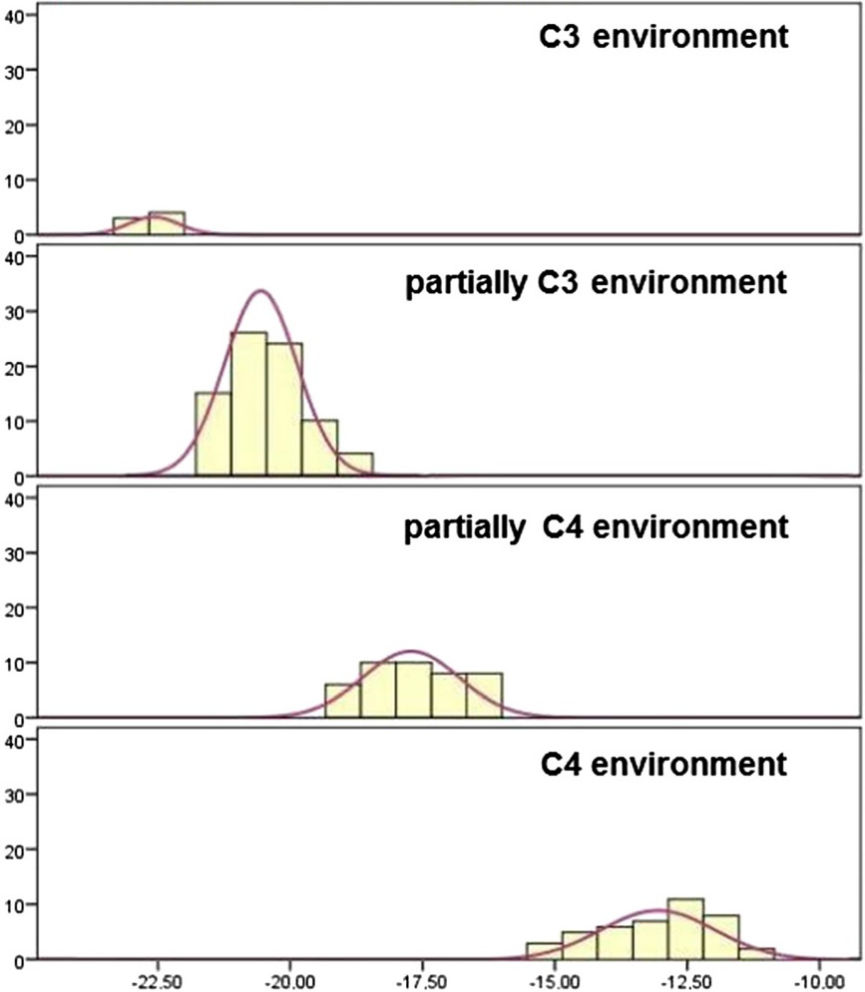
**Histogram displaying δ** ^**13**^ **C values in 170 rattle segments from the snakes studied, according to the distribution of their environments.** C_4_: C_4_ environment, PC_4_: partially C_4_ environment, C_3_: C_3_ environment, PC_3_: partially C_3_ environment.

**Figure 7:**
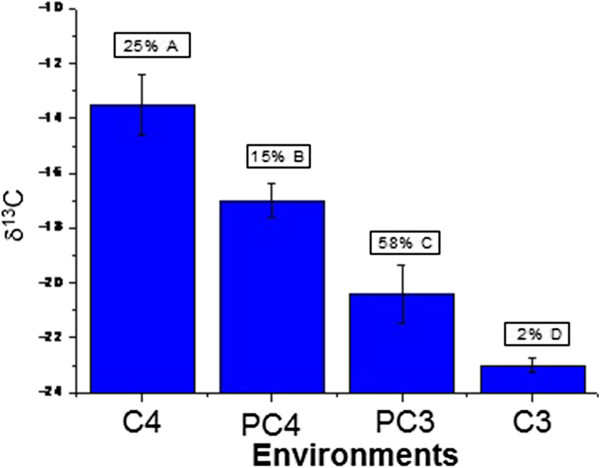
**Mean δ** ^**13**^ **C values and representation percentages of the 170 rattle rings analyzed, according to environment.** C_4_: C_4_ environment, PC_4_: partially C_4_ environment, C_3_: C_3_ environment, PC_3_: partially C_3_ environment.

**Figure 8:**
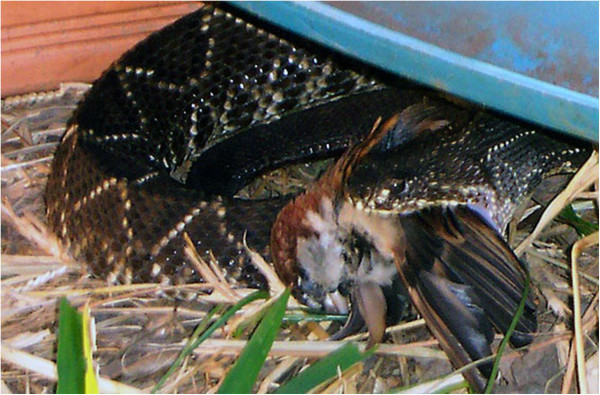
***Crotalus durissus terrificus*** **eating a sparrow (** ***Passer domesticus*** **) in CEVAP captivity.**

Figures 6 and 7 show the distribution of the δ^13^C values from 170 snake rattle segments, according to the environments C_3_, C_4_ and partially C_3_ and C_4_. Figure 8 shows a *Crotalus durissus terrificus* snake captured and fed a bird (*Passer domesticus*).

## Discussion

The isotopic profile (Figure 1) shows that, from birth to the development of the fourth segment (around 18 months of age), the first snake had consumed a diet based on animals living in an environment closely resembling C_4_. The fifth segment showed an isotopic value compatible with partially C_3_; in other words, there was a dietary change in prey that had fed on fruits, rice, soy, coffee and large trees. In the sixth segment the snake returned to eating animals from a strictly C_4_ environment, at which point it was captured and donated to CEVAP, initiating a controlled diet based on rodents from the animal house that consume solely balanced feed produced from a partially C_3_ environment. There was a change in the nature of the food energy source from the old environment (C_4_) to the captive (C_3_). These results are compatible with its life history, since this snake was captured in a maize crop – the representative plant of C_4_ environment.

Isotopic analysis of the profile of the rattlesnake 4 (Figure 2) shows that it was born in a mixed C_3_-C_4_ environment, where it remained for approximately six months and was then captured and taken to CEVAP. In captivity, it was offered live rodents that had the same isotopic profile of the ration they received. To complete the turnover of carbon, this animal had to be held in captivity with the same dietary energy supply for at least 33 months. Therefore, there is a slower turnover due to slow metabolism that provokes a slow rate of tissue production, which includes the rattle. These data agree with those by Mizutani *et al*. [5], which suggest that animals with a slow metabolism present a slow turnover, while those with an accelerated metabolism show a fast turnover.

Figure 3 shows the profile of the offspring from a mother recently captured and donated to CEVAP, indicating that both snakelets have the same isotopic initial values, namely −16.2‰. Both received since birth a diet of rodents from the animal house. Since it were animals in the growth phase, we found that the turnover was faster than that of adult animals acquiring, therefore, the isotopic equilibrium earlier. In this case, the first animal reached the equilibrium level at approximately 18 months and the second at around 28 months. The observation of these animals in the growth phase corroborated the study by Mizutani *et al*. [5], which showed that accelerated metabolism causes a rapid turnover. The isotopic values of both primary segments proves that they are from the same litter.

Figure 4 displays an analysis of the mothers and their offspring. The first mother was from a partially C_4_ environment while the second mother originated from a strictly C_4_ environment. The offspring of the first mother showed isotopic values from the same environment. These snakes were pregnant in nature when captured and donated to the IB. After a period of approximately one month, the first gave birth to three snakes and the second, five. All were born with the primary segment. Isotopic analysis of δ^13^C in mother rattlesnake 1 and its three offspring revealed that the last segment of the mother rattle was similar to those of the snakelets. Similar results were observed for mother rattlesnake 2 and its offspring. These findings show that the mother’s diet in nature can be traced in offspring rattles. There was also a small fractionation factor between mother and newborn snakes of around ± 1‰ for δ^13^C, which may suggest that the isotopic values of the primary segment of the offspring are close to those of the last segment of the mother.

Figure 5 shows that the majority of mice (88.23%) captured in the field were from a C_4_ environment. Regarding the second wild newborn snake, it was originally from a partially C_3_ environment and after migrated to a partially C_4_. The others were born in a C_4_ environment and remained in the same.

Figures 6 and 7 display a histogram and the mean values of δ^13^C in the 170 rattle segments of the studied snakes, according to the distribution of environments. It was verified that most of the values (58%) belong to an environment that is partially C_3_.

Additional file 1 shows two biochemical pathways of carbohydrate synthesis that carbon isotopes can follow in nature. The animals that feed on C_3_ and C_4_ plants will incorporate both types of isotopes. Thus, it may be concluded that *Crotalus durissus terrificus* snakes are predators of animals such as birds, mammals, reptiles and amphibians that had fed on C_3_ and C_4_ plants. The rattle rings enabled not only a retrospective analysis of the lifelong alimentary history but also speculation as to whether they were also eating birds, as can be observed in Figure 8, which provides a gain in the competition for survival in environments that are increasingly hostile and devastated. In relation to mice, it must be emphasized that those captured in a natural setting were predominantly consuming C_4_ plants. In captivity, these animals are fed ration produced from C_3_ plants, especially rice, soy and other legumes. Consequently, upon entering captivity, all the snakes alter their isotopic profile to that of a C_3_ environment.

Between 1993 and 1995 Sant’Anna and Abe [13] analyzed the stomach contents of 633 rattlesnakes (*C. d. terrificus*) in southeastern Brazil. The authors observed that the gastric and intestinal content was constituted mostly of the remains of rodents and small marsupials, and noted that newborn rattlesnakes fed primarily on rodents and presented the lowest rate of animals with stomach content [13]. It should be highlighted that on that occasion, no birds were found in the gastric content. The current observations show that these animals incorporated birds in their diet, as shown by Figure 8 recently taken in CEVAP captivity.

## Conclusions

Based on the results, we conclude that:

- ● *Crotalus durissus terrificus* eat animals whose diets comprise both C_3_ and C_4_ plants_._
- ● The rate of complete turnover of newborn snakes born in captivity until reaching the equilibrium level varies between 18 and 24 months.
- ● The rate of complete turnover of adults held in captivity until reaching the equilibrium level varies from 33 to 36 months.
- ● The primary rattle of newborn snakes reflects the mother’s diet during pregnancy, which can be confirmed by isotopic analysis of the last rattle segment the mother’s body.
- ● The ration produced in Brazil and offered as food to the rodents in captivity is produced essentially from rice, soy and other plants.
- ● It is possible to speculate that besides rodents, snakes may also be feeding on birds.
- ● One may also question whether this new behavior could alter the future composition of the venom of these snakes.
- ● Further studies are required to confirm all or some of these hypotheses.

## Electronic supplementary material

Additional file 1: **Biochemical pathways of carbon acquisition used by plants.** C_4_: C_4_ environment, PC_4_: partially C_4_ environment, PC_3_: partially C_3_ environment, C_3_: C_3_ environment. (PPTX 38 KB)
