# Supplementary material for: Does the rattle of Crotalus durissus terrificus reveal its dietary history?
Source: J Venom Anim Toxins Incl Trop Dis. 2014 Dec 9;20:53. doi: 10.1186/1678-9199-20-53 (PMC4276105; doi:10.1186/1678-9199-20-53)
Supplement: Supplementary file 1 — Additional file 1: Biochemical pathways of carbon acquisition used by plants. C4: C4 environment, PC4: partially C4 environment, PC3: partially C3 environment, C3: C3 environment. (PPTX 38 KB) [file 40409_2014_75_MOESM1_ESM.pptx]

## Slide 1
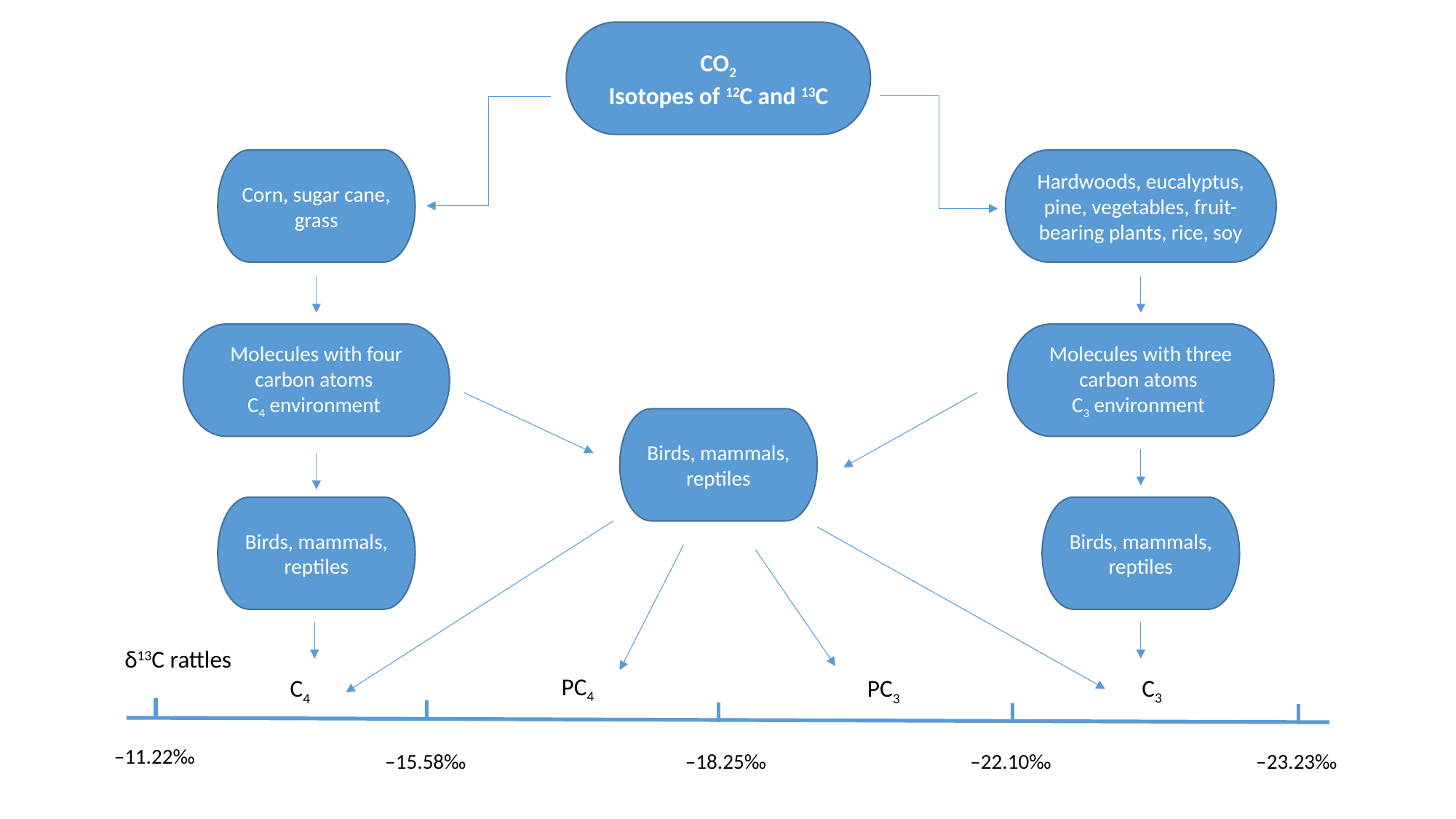

CO2
Isotopes of 12C and 13C
Corn, sugar cane, grass
Hardwoods, eucalyptus, pine, vegetables, fruit-bearing plants, rice, soy
Molecules with three carbon atoms
C3 environment
Molecules with four carbon atoms
C4 environment
Birds, mammals, reptiles
Birds, mammals, reptiles
Birds, mammals, reptiles
δ13C rattles
PC4
C3
C4
PC3
–11.22‰
–15.58‰
–22.10‰
–18.25‰
–23.23‰
